# Admissions for Atrial Fibrillation in the Elderly Population: Evaluation of Demographic Predictors of Mortality From the National Inpatient Sample

**DOI:** 10.7759/cureus.85634

**Published:** 2025-06-09

**Authors:** Youssef Jalloul, Joseph El Roumi, Hani Tamim, Moied Al Sakan, Shaker Eid, Marwan Refaat

**Affiliations:** 1 Internal Medicine, American University of Beirut Medical Center, Beirut, LBN; 2 Cardiology, Cleveland Clinic, Ohio, USA; 3 Clinical Research Institute, American University of Beirut Medical Center, Beirut, LBN; 4 Cardiology, American University of Beirut Medical Center, Beirut, LBN; 5 Internal Medicine, Hermann Memorial Health System, Texas, USA; 6 Cardiovascular Disease, American University of Beirut, Beirut, LBN

**Keywords:** atrial fibrillation, elderly, hospital bed size, location, race, region, sex, teaching status

## Abstract

Atrial fibrillation (AF) is the most common arrhythmia to affect elderly patients above 70 years of age and causes a significant burden. Multiple demographic factors have been linked to increased mortality in general in patients admitted with AF. The aim of our study is to delineate those factors in elderly patients admitted with atrial fibrillation in the United States. We used the Nationwide Inpatient Sample to evaluate the mortality of patients aged more than 70 admitted with AF between 2005 and 2014. We looked at variables such as race, sex, hospital location, hospital teaching status, hospital bed size, and hospital region to elucidate the association, if present, between those variables and mortality in our patient population. Specific ICD-9-CM codes were used to identify the study patients and their outcomes. Our results showed that 2,163,343 elderly patients above 70 years of age were admitted between 2005 and 2014, inclusive, with a diagnosis of AF. In those aged greater than 70, there was an association between sex and mortality (1.37% of females died vs. 1.48% of males died, p=0.004). However, patients who were white had a lower mortality rate than other races (1.38% of white patients died) at a p-value <0.0001. There was no correlation between hospital bed size or hospital teaching status (teaching vs. non-teaching) and mortality, with a p-value of 0.31 and 0.57, respectively. However, hospital location (1.39% mortality in urban vs. 1.51% in rural hospitals) and hospital region (the least mortality rate 1.24%, was found in Midwest and the highest, 1.58%, was found in Northeast), were associated with mortality, with corresponding p-values of 0.022 and <0.0001 respectively. The results of our analysis signify the need for further advancement of the hospital and regional-based resources. These outcomes reflect the gaps in the uniformity of nationwide medical care that the United States aims to attain.

## Introduction

Atrial fibrillation (AF) has been described as the epidemic of the present millennium [1]. It is considered the most commonly encountered arrhythmia in clinical practice. [2,3] Strokes related to AF are particularly serious; they tend to be more disabling and result in more deaths when compared to strokes from other causes [4,5]. AF is a major public health burden that disrupts the quality of life of affected patients and leads to substantial economic costs [6,7]. It is well known that AF affects more men than women, and its incidence doubles with every decade of life [8-10]. Although cardiovascular (CV) disorders promote AF incidence, Whites are at a heightened risk of developing AF in the absence of CV comorbidities compared to other racial groups [11]. While new medical and interventional therapies have been introduced in the care of patients with AF over the past two decades, the age-adjusted prevalence of AF is still on the rise [12, 13]. In fact, AF is projected to affect by the year 2050 more than 15.9 million individuals in the United States [12] and 17.9 million people in Europe by the year 2060 [14, 15]. 

To note that the overall prevalence of AF in our cohort is estimated to fall between 24-28.5%, which aligns with data from publications around the time of the study [8,9]. Recently, a report from Medicare beneficiaries highlighted that the prevalence of AF in the elderly showed an increasing yearly trend, such that the mean annual rise in magnitude from 1993 to 2007 was 4.3% among patients 66 to 69 years of age and 5.4% among patients 90 years or older [16]. Moreover, Freeman et al. demonstrated in a sample from the National Inpatient Sample (NIS) that adjusted rates of in-hospital mortality from AF decreased by 3.84% between 1999 and 2013 [17]. However, both 30-day and 1-year mortality increased from 3.9% to 4.9% and 13.7% to 16.4%, respectively [16]. This ultimately reflects the high prevalence of this condition and the overall health burden and functional impairment associated with this entity in the elderly population [18]. 

The aim of our study is to examine regional and hospital-based variables associated with mortality in a population of elderly patients admitted with a diagnosis of AF in the United States. As previously iterated, AF has higher prevalence, higher disease burden, and higher mortality as age increases. 

## Materials and methods

We extracted data from the National Inpatient Sample (NIS) database from 2005 to 2014. The NIS is a longitudinal hospital inpatient database, as part of a family of databases and software tools developed for the Healthcare Cost and Utilization Project (HCUP). The NIS is designed to produce US regional and national estimates of inpatient utilization, access, charges, quality, and outcomes, and it collects annual inpatient discharge data from over 1000 hospitals across the United States. The NIS approximates a 20% stratified sample of all discharges from US community hospitals, excluding rehabilitation and long-term acute care hospitals. The NIS contains information on all hospital stays, regardless of expected payer, for the hospital stay. Diagnoses in the NIS are specifically coded using standard International Classification of Diseases, Ninth Revision, Clinical Modification (ICD-9-CM) codes. Some variations and changes in the ICD-9 codes took place during this 10-year study period, which we have taken into account while collecting and analyzing the data. 

We identified all hospitalizations using ICD-9-CM diagnostic code 427.31 consistent with AF as the primary admission diagnosis and as the primary reason for hospital admission across 240 NIS strata. 

We provided a demographics table that contains specific ICD-9 codes in order to shed the light on the prevalence of the comorbidities that we looked at such as alcohol intake, smoking, hyperlipidemia, prior myocardial infarction (MI), prior percutaneous coronary intervention (PCI), prior coronary artery bypass grafting (CABG), prior stroke or transient ischemic attack (TIA), and acute stroke amongst our patient population (Table 1). Furthermore, we looked at other variables such as age, race, sex, hospital location, hospital teaching status, hospital bed size, and hospital region in order to elucidate their influence on mortality over a 10-year period. 

**Table 1:** ICD-9 comorbidities MI - myocardial infarction; PCI - percutaneous coronary intervention; TIA - transient ischemic attack; ICD-9 - International Classification of Diseases Ninth Edition

Comorbidity	ICD-9 Codes					
Smoking	305.1	305.1	305.11	305.12	305.13	V15.82
Hyperlipidemia	272	272.1	272.2	272.3	272.4	
History of MI	412					
History of PCI	V45.82					
History of CABG	V45.81					
History of stroke or TIA	V12.54					
Acute stroke	433.01	433.11	433.21	433.31	433.81	433.91
	434.01	434.11	434.91	437.1	436	

Annual rates of AF were calculated between 2005 and 2014 per 100,000 US population. Data was stratified according to the NIS strata and year of discharge, and clustered based on hospital ID. The NIS database provides "trend weights" to facilitate analysis of trends across multiple years and to obtain national estimates. Samples were then weighed according to discharge weight or trend weight, depending on the year of the NIS sample. 

Statistical analysis was performed using SPSS version 23 (IBM Inc., Armonk, New York). Complex sample analysis was used to calculate the frequency and percentage count of each variable of interest. Univariate logistic regression models were constructed to calculate the odds ratio of the association between mortality due to AF in the elderly above the age of 70 according to sex, race, hospital bed size, hospital location (rural or urban), and hospital state region. Graphs and tables were generated in order to elucidate this correlation as well. Multivariate logistic regression was also generated based on the built model. 

## Results

Two million, one hundred sixty-two thousand, four hundred fourteen admissions for AF in the United States between 2005 and 2014 were identified in elderly patients defined as those above the age of 70. Demographic and clinical characteristics are shown in Tables 2-3.

**Table 2:** Baseline characteristics of elderly patients admitted with atrial fibrillation, National Inpatient Sample 2004-2014

Variable	Sub-variable		Count, n=2,162,414	Percentage
Total age group	71-75		527,105	24%
	76-.80		560,268	26%
	81-.85		534,249	25%
	86-90		432,766	20%
	>90		108,026	5%
Sex	Male		774,533	36%
	Female		1,387,427	5.40%
Race	White		1,607,790	87%
	Greek		89,278	5%
	Hispanic		83,877	5%
	Asian or Pacific Islander		24,747	1%
	Native American		8008	0%
	Other		35,293	2%
Primary payer	Medicare		1,997,435	92%
	Medicaid		18,827	1%
	Private insurance		121,894	6%
	Self-pay		7451	0%
	No charge		842	0%
	Other		13,644	1%
Hospital region	Northeast		460,604	21%
	Midwest		542,096	25%
	South		832,098	38%
	West		327,615	15%

**Table 3:** Baseline characteristics of elderly patients admitted with atrial fibrillation, National Inpatient Sample 2004-2014 PCI - percutaneous coronary intervention; CABG - coronary artery bypass grafting; TIA - transient ischemic attack

Variable	Sub-varaible	Count, n=2,162,414	Percentage
Hospital division	New England	37,260	6%
	Middle Atlantic	105,385	16%
	East-North Central	116,885	17%
	West-North Central	47,700	7%
	South Atlantic	148.385	22%
	East-South Central	50,515	7%
	West-South Central	67,475	10%
	Mountain	34,815	5%
	Pacific	66,840	10%
Hospital control	Government, non-federal	155,670	10%
	Private, non-profit	1,188,720	76%
	Private, invest owned	227,065	14%
Hospital location and teaching status	Rural	337,925	16%
	Urban nonteaching	946.07	44%
	Urban teaching	869,178	40%
Hospital bed size	Small	321,678	15%
	Medium	552,875	26%
	Large	1,278,619	59%
Social history	Alcohol	29,238	1%
Comorbidities	Smoking	358.085	17%
	Hypertension	1,518,530	70%
	Diabetes mellitus	503934	23%
	Congestive heart failure	9706	0%
	Peripheral vascular disorder	180,874	8%
	Valvular disease	4884	0%
	Pulmonary circulation disorder	2565	0%
	Chronic lung disease	499,718	23%
	Renal failure	316,975	15%
	Anemia	324,581	15%
	Liver disease	17,382	1%
	Obese	139,647	6%
	Hyperlipidemia	937,051	43%
	History of myocardial Infarction	168,907	8%
	History of PCI	172,227	8%
	History of CABG	196,719	9%
	History of stroke/TlA	143,263	7%
	Acute stroke	18,052	1%

The mean age was 81, and the majority of patients were females (1,387,427 or 64%) compared to males (774,633 or 36%). Of the patients, 560,268 (26%) were in the 76 to 80-year subgroup, and 108,026 (5%) were in the very elderly subgroup defined as above the age of 90. Eighty-seven percent of patients were White, followed by Black (5%) and Hispanic (5%). Medicare was found to be the primary payer (92%) involved in the financial coverage of this population of elderly patients, followed by private insurance payers (6%). Ninety percent of hospital admissions due to AF were cared for in private hospitals. Seventy percent of patients were hypertensive, 43% had dyslipidemia, 23% were diabetics, 7% had a history of stroke or transient ischemic attack, and 18,052 patients (1%) were admitted with AF-causing acute embolic stroke. Six percent of patients were obese, 8% had previous myocardial infarction, 8% had previous coronary artery bypass grafting surgery, and 8% had previous percutaneous coronary intervention. Fifteen percent had chronic kidney failure and anemia, and 23% had a chronic lung disorder. 

**Figure 1:**
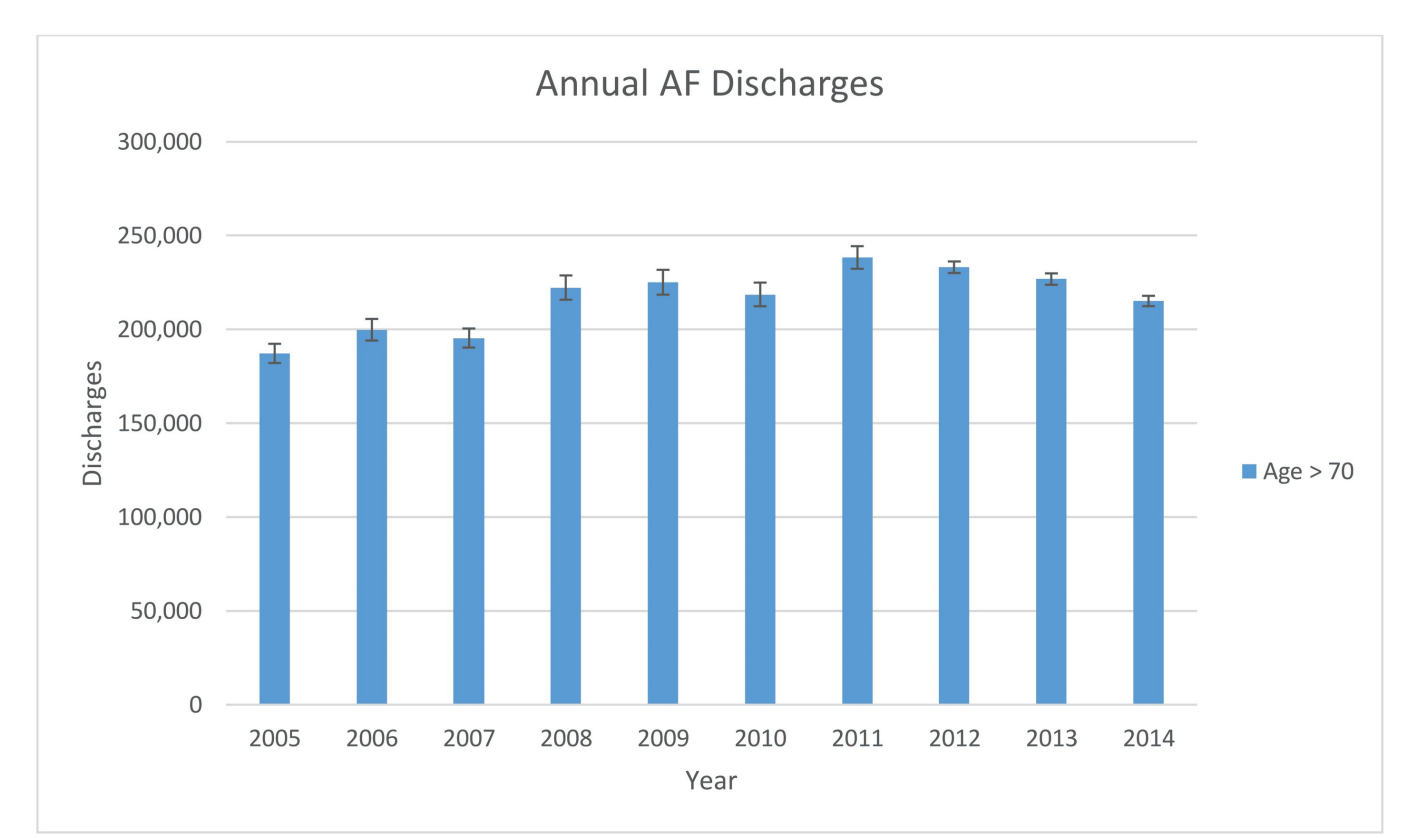
Annual AF elderly patients discharges by year AF - Atrial fibrillation

Figure 1 reflects trends in annual AF discharges in elderly patients and shows a rise in AF admissions by 15% between 2005 and 2014 and a peak incidence rate in AF cases in 2011, with 215,200 new cases. Cumulative mortality due to AF from 2005 to 2015 was found to be at 21,000 cases (1.4% of total cases with primary diagnosis of AF were deceased at discharge level). 

Of the admissions, 1,278,619 (59%) were recorded at large bed size hospitals, 552,875 (26%) in medium bed size hospitals, and 321,678 (15%) in small bed size hospitals. Of the AF admissions, 832,098 (38%) were received in hospitals located in the South USA (38%), of which 148,385 admissions were in the Southern Atlantic (22%), followed by 542,096 (25%) in the Midwest. Admissions that took place in hospitals located in a rural region were 337,925 (16%), while the rest occurred in hospitals located in an urban region. Urban admissions were almost equally distributed between teaching and non-teaching hospitals (40% and 44%, respectively). 

Of the patients hospitalized with AF, 30,391 patients (1.4%) died during their hospitalization. 

**Figure 2:**
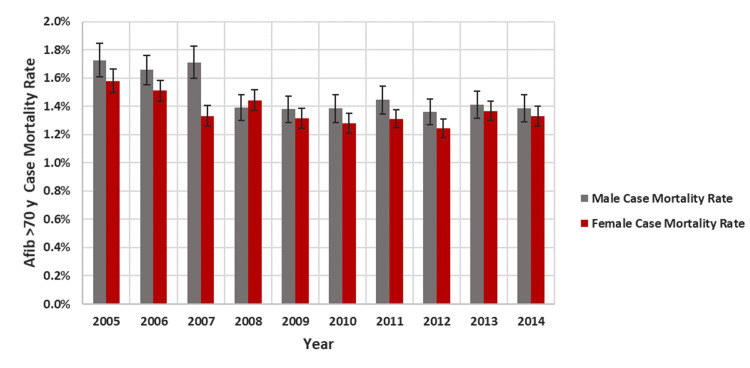
Case mortality rate of AF patients with age more than 70 according to sex and year AF - atrial fibrillation

Figure 2 outlines the case fatality rate of males in comparison to females across the years and delineates a direct association between sex and mortality (1.48% of males died and 1.37% of females, p=0.004). 

Table 4 outlines the mortality of elderly patients according to race. White elderly patients had the lowest mortality, and Black elderly individuals had the highest mortality according to race (1.38% for White and 1.93% for Black patients, p-value < 0.0001). 

**Figure 3:**
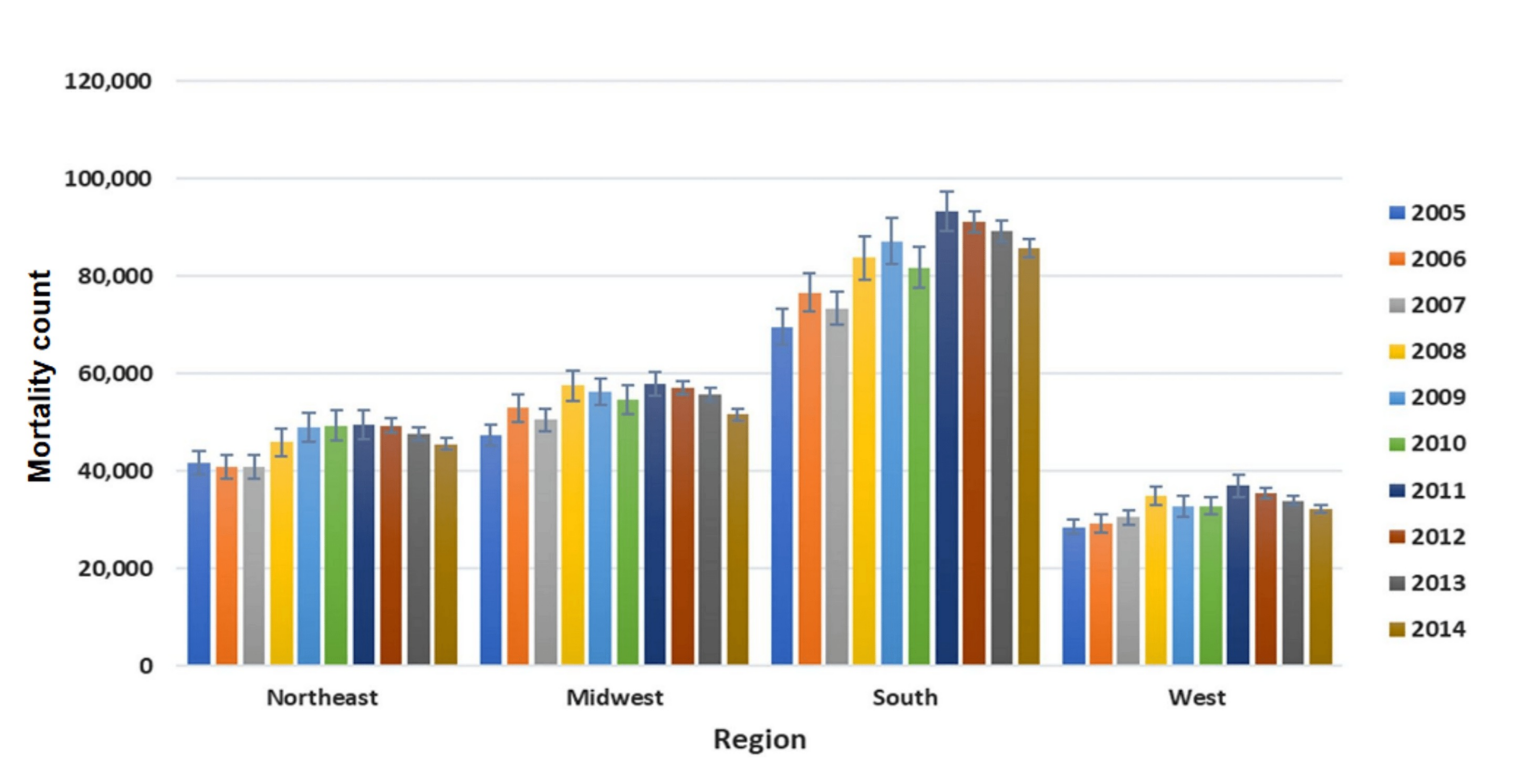
Annual AF mortality count by year and state region AF - atrial fibrillation

In addition, hospital location and hospital state region were both identified as independent predictors of mortality in this population (1.39% mortality rate in urban compared to 1.51% in rural hospitals, the lowest mortality rate of 1.24% was found in the Midwest compared to the highest, 1.58% in the Northeast) with corresponding p-values of 0.022 and <0.0001 respectively (Figure 3). 

**Table 4:** Association between hospitalization due to AF and specific race of elderly patients above the age of 70 AF - atrial fibrillation

Race	Hospitalization outcome		Total
	Survived	Deceased	
White	1,585,111	22,148	1,607,259
	98.62%	1.38%	
Black	87,513	1727	89,240
	98.10%	1.99%	
Hispanic	82,586	1260	173,088
	98.50%	1.50%	
Asian or Pacific Islander	24,317	438	24,756
	98.22%	1.78%	
Native American	7884	123.86	8008
	98.45%	1.55%	
Other	34,780	511	35291
	98.55%	1.45%	
Total	1,822,193	26,211	1,822,219
	98.60%	1.40%	

Interestingly, as shown in Table 5, hospital bed size did not influence mortality, and its association with mortality due to AF was found to be statistically non-significant (p-value 0.335).

**Table 5:** Association between mortality and hospital bed size (small, medium, large) 9528 patients above the age of 70 had a missing hospital bed size variable in the Healthcare Cost and Utilization Project (HCUP) National Inpatient Sample (NIS) database

Hospital bed size	Hospitalization outcome		
	Survived	Deceased	Total
Small	316,989	4291	321,280
	98.60%	1.40%	
Medium	545,078	7883	552,962
	98.57%	1.43%	
Large	1,260,557	18,087	1,278,644
	98.60%	1.40%	
Total	2,122,624	30,262.60	2,152,886

## Discussion

The aging population in the US is projected to rise from 40.2 million to 88.5 million people by the year 2050 [19]. Enhanced detection of AF and improved survival from cardiovascular diseases have contributed to an expanding number of patients diagnosed with AF [18,20-22]. In fact, an aging population with high prevalence of AF-predisposing risk factors such as obesity, coronary artery disease, diabetes mellitus, and chronic obstructive pulmonary disease has provided the appropriate substrate for AF development and maintenance in this patient population [18,20-22]. 

Mortality from AF is reported to be higher in the elderly compared to younger age groups, mainly due to both the increased risk of stroke and other comorbidities in the elderly, in addition to an increased risk of bleeding conferred by oral anticoagulant intake [23-26]. Furthermore, a study by An et al. supports the concept that AF-related mortality may be effectively reduced not only by AF management but also by strict optimization and treatment of both cardiovascular and non-CV comorbid conditions [27]. 

AF and sex differences

Vasan et al. reported a statistically nonsignificant slight increase in AF-related mortality in women and concluded that death rates between both sexes are equivalent over time. The group chronicled a cumulative annual increase in the rates of death from AF by 11.2% between 1991 and 2015 in both males and females [28]. Conversely, a study by Magnussen et al. has negated the association between AF-related mortality and sex, thus reporting a >3.5-fold increase in mortality for both sexes across the years [3]. The results of our study are discordant from those described by previous studies, whereby we found a statistically significantly higher mortality in elderly males admitted with AF compared to their female counterparts. We also describe higher incidence cases of AF in females than in males, as opposed to previously published literature. We hypothesize that this variation can be mainly attributed to three reasons. Firstly, novel oral anticoagulant (NOAC) use compared to adequately controlled anticoagulation with warfarin has been associated with lower rates of intracranial hemorrhage, a life-threatening condition, in females but not in males [29]. Unfortunately, the HCUP databases do not record medication intake of the patients, including oral anticoagulation agents, in order to ascertain this hypothesis. Secondly, age-dependent comorbid conditions such as obstructive sleep apnea (OSA), a famous driver of AF in the elderly, have a male-to-female preponderance of three-to-one, and prevalence data show that males are more severely affected by OSA than females [30]. Furthermore, increasing survival of elderly patients has translated to a 35% prevalence of obesity amongst this population. It is well known that obesity provides the homeostatic milieu for the perpetuation of the metabolic syndrome and worsens the cardiovascular status of the patient, leading to a higher mortality from cardiovascular causes, AF being a culprit as well [31]. It is noteworthy to mention the counterintuitive "obesity paradox" where patients with obesity and hospitalization for AF have a better prognosis and lower mortality than non-obese cohorts [32-35]. Other comorbidities associated with AF, such as diabetes mellitus and cigarette use, have a higher proportion of affected males compared to females in the elderly subgroups [36,37]. 

AF and race 

While a higher incidence of AF was observed in White compared to Black patients in the ARIC study, it was also shown that Black participants had considerably greater risk of developing adverse outcomes due to AF [2]. Conversely, a large study in 2006 contemplating Medicare beneficiaries identified that Black individuals with AF have twice the risk of stroke compared to White individuals [38]. Some have suggested that this increased stroke and mortality risk extends beyond the low rates of anticoagulant use in Black patients and may be mainly related to poor cardiovascular control of AF-enhancing factors such as obesity, diabetes mellitus, heart failure, and hypertension [2,39-42]. Our study simply reinforces the results published by the ARIC study, that White patients have a higher prevalence of AF, but Black patients have higher AF-related mortality, possibly caused by poor control of other CV comorbidities. 

AF and hospital location (urban and rural) 

In 2013, it was estimated that the probability of a patient dying in a rural hospital was 10% higher than in an urban hospital [43]. It has been reported that the elderly residing in rural communities have a higher disease burden and less access to healthcare than their urban counterparts [43]. Elderly people residing in rural areas participate in activities such as consuming unhealthy diets and smoking that increase their risk of developing AF, compared to those living in urban areas [44-47]. Moreover, elderly people, based on where they live, face a multitude of economic and geographic barriers to health, thus widening a so-called rural-urban disparity in mortality, medical care, and life expectancy [48-50]. This discrepancy in outcomes has been previously studied and examined by Viallpiano et al. in 2017 [43]. In fact, rural health system characteristics may explain the variability of hospital admissions and survival of patients. For example, the number of doctors per capita, the quality of provided care, and restricted access to hospice services may predispose patients to die in rurally located hospitals compared to urban hospitals [51]. Our results go hand in hand with the aforementioned studies, whereby we demonstrated that rural community elderly who were admitted with AF had an 8.6% increased mortality when compared to urban-residing elderly. However, Hall et al. suggested another explanation for this regional gap in healthcare. This group mentioned a growing tendency of urban residents to bypass local hospitals and seek healthcare in urban hospitals [52]. However, the way in which this bypass trend skewed mortality towards rural hospitals has not been clearly established. Eventually, a rural hospital's quality of care cannot be the only factor to blame when it comes to increased rates of mortality in the rural setting. These perplexing hypotheses require more dedicated research and credibility before drawing nationwide conclusions about this matter. 

AF and state region 

During the 1960s, the term "stroke belt" was coined to describe the southeastern states of the US [53-55]. In these states, higher rates of strokes were diagnosed as compared to other regions in the country, and were associated with significant mortality, defined as more than 10% age-adjusted mortality above the US-average according to the National Heart, Lung and Blood Institute [53, 56]. More recently, regional variation in AF-related mortality was observed, with higher rates in states such as Columbia, Kentucky, Alabama, and West Virginia [28]. 

Claxton et al. examined geographic variations in stroke rates in patients with AF in order to better evaluate state-level strategies for AF management in regions considered to be at elevated stroke risk [5]. They concluded that stroke rates did not distribute according to the previously described stroke belt in the US and found no association between oral anticoagulant use for AF and regional distribution of stroke mortality rates [5]. This study suggests that differences in stroke rates are caused by risk factors other than AF, such as uncontrolled hypertension and poor socioeconomic status that negatively impact survival [5,57]. 

Our results show the highest mortality from AF in elderly patients residing in the Northeastern states and the least mortality in those residing in the Midwest. Considering the elevated mortality associated with AF-related stroke, our study reinforces the published results of Claxton et al. in abolishing the southern US stroke belt myth. Although we could not directly ascertain the direct cause of death of those afflicted with AF (whether from stroke, uncontrolled hypertension, or other factors), AF-related mortality in the elderly follows a geographic distribution that warrants further studies and investigation. 

AF and coronary artery disease (CAD)

One important consideration to note is that underlying coronary artery disease (CAD) remains one of the most critical and prevalent risk factors for atrial fibrillation (AF) in the elderly, accounting for up to 70% of AF cases in this population [58]. Recent studies emphasize that the coexistence of CAD not only increases the risk of developing AF but also significantly worsens its prognosis. Patients with both conditions face higher rates of thromboembolic events, heart failure, and mortality compared to those with AF alone [59]. The shared pathophysiological mechanisms, such as endothelial dysfunction, inflammation, and atrial remodeling, further compound the clinical burden [60]. These findings underscore the importance of early CAD assessment in elderly AF patients and support a more integrated approach to management [61,62].

What sets our study apart is its nationwide scope and its focus on elderly patients with AF across diverse geographic and demographic strata. While previous studies have highlighted regional disparities in stroke and AF-related outcomes, our analysis uniquely quantifies the mortality gap between rural and urban settings and across US state regions, using a large, contemporary dataset. Unlike earlier research that often focused on isolated populations or specific comorbidities, our study integrates multiple layers of risk - age, sex, race, comorbid conditions, and hospital location - into a unified analysis. This allows for a more nuanced understanding of how structural and systemic factors contribute to AF-related mortality. By doing so, we provide actionable insights that can inform targeted public health interventions and resource allocation strategies, particularly in underserved regions such as the rural South and the historically defined "stroke belt".

Limitations 

This study has a few limitations that deserve to be systematically mentioned. First of all, this study is a retrospective cross-sectional study that tracks hospitals but not patients; thus, it is limited in terms of drawing direct correlations and causality. Also, diagnosis of cases with AF is based on diagnoses recorded strictly at discharge and not upon admission. Hence, unified national diagnostic criteria for AF inclusion lack ECG data, duration of AF, and AF subtype. Secondly, this database covers around 95% of US admissions, with 5% of admissions that are missed and not recorded in the database. Thirdly, this study entails the US population only, and its results cannot be extrapolated to other regions of the world with different ethnic groups and sociodemographic differences, not to mention the variability in health care systems and policies. Lastly, repetitive patient discharges for the same patient with the sampling criteria may have occurred and are accounted for as a new discharge diagnosis in the database set. 

## Conclusions

AF is a signature of cardiopulmonary diseases in the elderly and imposes higher morbidity and mortality. Many social and demographic factors, such as sex, race, region, and hospital factors, appear to independently influence mortality rates in those above the age of 70. It is imperative to strive for unified care across the whole of the United States in order to minimize segregation of care between elderly of different sex, region of residence and race, by implementing local and national strategies that reinforce oral anticoagulation when medically indicated and aim at controlling CV and non-CV risk factors that favor AF initiation and maintenance. 
